# Annotations

**Published:** 1898-12-17

**Authors:** 


### ANNOTATIONS.

### The University of London and the Imperial Institute.

It is distinctly unfortunate that the offer made by the Government to arrange for the housing of the new University of London in the Imperial Institute at South Kensington should have been associated by some with the suggestion that the name of the University should be changed to that of the Imperial University of London. Much of the difficulty which has hung over this question for so many years has really had its root in the fact that the title, University of London, was already appropriated by a body catering exclusively for unattached students. The rights of the University of London have been considered even to the verge of spoiling the new institution as a truly London University. This has been done to a large extent because the University of London, notwithstanding its name, has really been an Imperial degree-giving concern, and it was properly enough thought that, whatever happened, it would not be just to interfere with its long-standing relation to external students. On the other hand, it was decided that it would not be wise to found a new university with a new name for London alone, such as the suggested Gresham University. Hence the amalgamation. It must be understood, however, that the recent Act is the outcome of a demand for a local University for London, not an Imperial affair, and that the complications of the present scheme are the outcome of a willingness of its promoters to put up with much, so only that the institution in connection with which London students shall receive their education and their degrees shall be the University of London. To turn round now and dub it the Imperial University of London would, we think, be a breach of faith.

### A Friendly Warning.

At a recent meeting of the Hospital Board at Cape Town, South Africa, at which the question of the reception of consumptive patients was under discussion, Dr. Anderson, the house physician, said that a large number of consumptives came to the hospital in the last stage of the disease, and he found it hard to send them away. Many unfortunate persons who arrived by steamer landed in a penniless state. At present, he said, there was a little boy in one ward dying of phthisis, who had been sent out by his friends with only ten shillings in his pocket. He thought letters should be written to the home papers, stating very strongly that it was most inadvisable that such cases should be sent out. Another speaker pointed out the friendless, exhausted, and almost lifeless condition in which consumptives too often arrive at the Cape. We quite agree. There can be no doubt that the ill-advised rush of hopeless consumptives to places which have got a name for curing the disease is a matter approaching a public scandal. It is, however, a thing that is very difficult to check. These desperate cases are not, we fancy, as a rule, sent out by medical men. They are patients who, buoyed up by the eternal hopefulness of the consumptive, and tempted by stories of unexpected cures, determine to brave everything, and stake their all on one final cast for health. These patients are difficult to argue with, especially in view of the occasional occurrence of very extraordinary recoveries; and, indeed, the more strongly the hopelessness of a case may be urged the more fixed do these patients sometimes become in their resolve to make a rush for sunshine, and to take their chance. The medical profession should not then be blamed for all these sad catastrophes. At the same time, it must be admitted that the wide popularisation of medical topics in the lay press, and especially the public proclamation by leading medical men of a "crusade" against tuberculosis, which has almost for its watchword "Sunshine and fresh air," have led many patients to imagine they "know all about it," and have made it very difficult for medical men to exercise any control over their movements. We say that for a patient to go out to South Africa with fair prospect of cure the disease should not be far advanced and should not be in an acute or febrile state, while he should retain such appetite and digestion as not to be dependent on delicacies, should have strength enough to carry him to the elevated districts inland, and enough money to support him comfortably. This is safe advice, and good. But every now and then cases occur which do not conform to these neat regulations and still get well. These are the cases which the consumptive hears of and acts upon. It is a pity, and we are sorry; but we do not wonder. Perhaps we should do the same.

### A Collapsible Bath for Use in Bed in the Treatment of Pyrexia.

Dr. Constantine MacGuire has described in the New York *Medical Record* a portable india-rubber bath-tub which he has devised for use in cases of pyrexia. The idea is not new, and, in fact, it has been carried out before in various ways, notably by turning up the sides of a large mackintosh sheet, making them rigid by rolling them over broom-handles, and folding up the corners so as to prevent the escape of the water; but those who can obtain Dr. MacGuire's bath will find it much more convenient than any such rough-and-ready plan. This new collapsible bath-tub consists of an india-rubber sheeting with an inflatable border which, when blown up, forms water-tight sides and ends to the bath. The bath is, in fact, much the same as the ordinary inflating camp bath, but is made to take a patient lying at full length. The air chamber may be inflated by a bicycle pump or, preferably, from a cylinder of compressed air, the process of inflation, when the cylinder is used, taking less than a minute. The discharge tubes, which are connected with the bath at the part which is depressed to the lowest point by the weight of the patient, will also empty the bath in less than a minute. At the termination of the bath, a dry sheet can be spread over the collapsed tub, and the patient can be made comfortable, or the tub can be removed in the same manner that an exchange of sheets is made. There can be little doubt that, where the tub has to be employed frequently, a collapsible arrangement such as is described will very materially facilitate its use, and will save the patient many of the risks of the bath treatment of pyrexia, especially in private practice where plenty of skilled assistance is not always available.

